# Incidence, prevalence and mortality of anorexia nervosa in individuals with childhood-onset type 1 diabetes: a nationwide retrospective cohort study in Sweden

**DOI:** 10.1136/bmjopen-2025-109015

**Published:** 2026-02-12

**Authors:** Magnus Sjögren, Erling Englund, Axel Åke Elias Erlandsson, Anna Möllsten

**Affiliations:** 1 National Highly Specialized Care for Severe Eating Disorders, Region Västernorrland, Sundsvall, Sweden; 2 Clinical Science, Psychiatry, Umeå university, Umeå, Sweden; 3 Department of Patient Safety, Development and Research, Region Västernorrland, Sundsvall, Sweden; 4 Umeå universitet Klinisk vetenskap, Umea, Sweden; 5 Department of Clinical Sciences, Pediatrics, Umeå University, Umeå, Sweden

**Keywords:** Mortality, General diabetes, Child & adolescent psychiatry, Eating disorders, EPIDEMIOLOGY

## Abstract

**Objectives:**

To investigate the incidence, prevalence and mortality of anorexia nervosa (AN) among individuals with childhood-onset type 1 diabetes (T1D) compared with matched controls in Sweden.

**Design:**

Retrospective nationwide cohort study using linked registry data.

**Setting:**

Nationwide, Sweden; population-based registers (covering the period 1977–2019).

**Participants:**

12 202 individuals diagnosed with T1D before age 15 years (5618 females; 6584 males) and 48 484 age-matched, sex-matched and municipality-matched controls without diabetes (23 618 females; 24 866 males).

**Primary and secondary outcome measures:**

AN diagnoses (International Classification of Diseases-10 codes F50.0 and F50.1) identified via the National Patient Register. Outcomes were period prevalence, point prevalence at ages 15 and 20 years, 10-year incidence rates and proportional mortality ratios (PMR), stratified by sex. ORs and incidence rate ratios (IRR) with 95% CIs were estimated using Mantel-Haenszel methods; Kaplan-Meier analysis compared time to AN diagnosis between groups.

**Results:**

The period prevalence of AN among females with T1D was 1.9% compared with 1.1% in controls (OR 1.64, 95% CI 1.31 to 2.06; p<0.001). The 10-year incidence rate for females with T1D was 74.7 per 100 000 person-years vs 45.2 per 100 000 person-years in controls (IRR 1.77, 95% CI 1.35 to 2.32). Point prevalence at age 15 years was 0.87% (T1D) vs 0.53% (controls) (IRR 1.65, 95% CI 1.16 to 2.35), and at age 20 years was 1.73% (T1D) vs 1.11% (controls) (IRR 1.55, 95% CI 1.20 to 1.99). The PMR for females with both T1D and AN compared with controls without either condition was 20.4 (95% CI 6.6 to 47.6). Male cases were few (n=4 in the T1D group; n=12 in controls).

**Conclusions:**

Females with childhood-onset T1D in Sweden have an elevated risk of AN and markedly higher mortality when both conditions are present. Despite the increased relative risk, the absolute risk of AN in females with T1D remained below 2%. These findings support routine screening for eating disorders in the T1D population, particularly among adolescent and young adult females.

**STRENGTHS AND LIMITATIONS OF THIS STUDY**

- Nationwide registry-based design with comprehensive population coverage and long-term follow-up (mean 12.5 years) ensures high external validity.
- Use of validated Swedish national registers with documented high diagnostic accuracy for both type 1 diabetes (T1D) and anorexia nervosa (AN) diagnoses.
- Matching on age, sex and municipality of residence in a 1:4 ratio reduces potential confounding from demographic factors.
- Registry-based diagnostic data may underestimate true AN prevalence due to under-reporting and cases managed exclusively in outpatient primary care settings.
- Low absolute numbers of male AN cases (n=4 in T1D group) limit statistical power for sex-stratified analyses and generalisability to males.

## Introduction

 Type 1 diabetes (T1D) is one of the most common chronic diseases with onset in childhood. In Sweden, approximately 1000 children are diagnosed with T1D annually, corresponding to a cumulative incidence approaching 0.9% by age 18 years in recent birth cohorts,^1^ with recent studies indicating even higher incidence rates.^2 3^ While T1D can also develop in adulthood, the present study focuses on childhood-onset disease. T1D carries substantial risk of both acute complications, such as diabetic ketoacidosis, and long-term sequelae including retinopathy, nephropathy and increased risk of cardiovascular disease. To reduce the risk of complications, optimal metabolic control is important and requires careful balance of carbohydrate intake and insulin administration.

Anorexia nervosa (AN) is a severe psychiatric disorder characterised by restrictive food intake, intense fear of weight gain, body image disturbance and significantly reduced body weight. The mortality rate in patients with AN is among the highest of all psychiatric conditions, approximately six times that of the general population, often due to suicide or organ failure secondary to malnutrition.^4 5^ The co-occurrence of T1D and AN presents particularly complex clinical challenges. Behaviours characteristic of AN—such as self-induced vomiting, excessive exercise and intentional insulin omission (sometimes termed ‘diabulimia’)—can lead to severe hyperglycaemia, ketoacidosis and accelerated development of diabetic complications.^6^ Furthermore, psychiatric comorbidities are common in AN and further worsen prognosis.^7 8^

### Prior epidemiological evidence and knowledge gaps

Several studies have examined the association between T1D and eating disorders. A Danish population-based cohort study by Dybdal *et al*^9^ found that children diagnosed with T1D had a twofold to fourfold increased risk of developing eating disorders, particularly AN. A meta-analysis by Mannucci *et al*^10^ reported no significant difference in AN prevalence between individuals with T1D and controls, but this analysis was limited by small sample sizes, heterogeneous methodology and data from over two decades ago when awareness and diagnostic practices for eating disorders were less developed. More recent systematic reviews and meta-analyses have reported higher prevalence of eating disorders in T1D populations, likely reflecting both improved recognition and true increases in incidence.^11 12^ A recent systematic review and meta-analysis found a pooled OR of approximately 2.4 for eating disorders in individuals with T1D compared with controls.^13^

Despite accumulating evidence, several important gaps remain. First, most prior studies have examined eating disorders as a broad category rather than focusing specifically on AN, which has distinct clinical features and outcomes. Second, many studies have relied on self-reported questionnaires or small clinical samples, limiting generalisability. Third, population-level data on mortality outcomes in individuals with concurrent T1D and AN are scarce.

### Study rationale

The present study addresses these gaps by using comprehensive Swedish national registers to investigate the incidence, prevalence and mortality of AN specifically among individuals with childhood-onset T1D compared with matched population controls. The Swedish registers offer high coverage, validated diagnoses and long-term follow-up, enabling robust epidemiological analysis. Understanding the magnitude of risk and mortality burden is essential for informing screening practices, clinical management and resource allocation for this vulnerable population.

## Research design and methods

### Study design and population

All children diagnosed with T1D before age 15 years and registered in the Swedish Childhood Diabetes Register (SCDR) from 1 July 1977 onwards were included. For the present analysis, we included individuals diagnosed between 1 January 1997 and 31 December 2019 to ensure that all AN diagnoses were coded using International Classification of Diseases, Tenth Revision (ICD-10) criteria (introduced in Sweden in 1997), thereby enhancing diagnostic consistency. To increase the probability of correctly identifying AN cases and reduce the inclusion of transient eating concerns, only individuals aged 12 years or older at any point during follow-up were included in the analysis.

From Statistics Sweden (Statistikmyndigheten SCB), four control individuals per diabetes case, matched by month and year of birth and municipality of residence, have been included to enable comparisons with a non-diabetes control population. Information on registered diagnoses according to the ICD-10 has been added via the National Patient Register at the National Board of Health and Welfare (Socialstyrelsen). The ICD-10 codes for AN (F50.0) and atypical AN (F50.1) were merged as they represent the same psychopathological spectrum and cross-over from one group to the other is very common.^14 15^

### Handling of missing data and censoring

All individuals were followed from the date of T1D diagnosis (or corresponding index date for controls) until first AN diagnosis, death, emigration or end of the study period (31 December 2019), whichever occurred first. Individuals lost to follow-up due to emigration or death were right-censored at the date of their last registered contact in Swedish national registers. The Swedish national registers have exceptionally high coverage (>99% for vital status and emigration), resulting in minimal loss to follow-up. Missing data on key covariates (age, sex, municipality) were absent due to the structure of the matching process. For clinical variables derived from the SCDR, coverage exceeded 98% for all key variables. Complete case analysis was therefore employed, and no imputation methods were used. Sensitivity analyses restricting to individuals with complete covariate data confirmed robustness of the main results (data available on request).

### Patient and public involvement

No patients or members of the public were involved in the design, conduct, or reporting of this retrospective registry-based study. The study was conducted using anonymised data from national health registers, and as such, there was no direct patient or public involvement. Results will be disseminated through scientific publication and may also be shared with relevant clinical and patient organisations as appropriate.

### Statistical basis

All analyses were done using SPSS (V.28.0, IBM).

Prevalence and incidence rate of AN among persons with T1D compared with the control population were calculated, both period prevalence (for the entire study period) and point prevalence of AN (at 15 and 20 years of age), stratified by sex.

The study included individuals diagnosed with T1D between 1 January 1997 and 31 December 2019, along with their respective matched controls (1:4). The starting date was chosen to include only individuals diagnosed with AN since ICD-10 was introduced. To increase the probability of a correctly set diagnosis of AN, only individuals older than 12 years were included. Controls were matched to cases based on age, sex and observation time (1:4).

### Statistical methods

Odds ratios (OR) and p values were calculated using Mantel-Haenszel’s common OR estimate with 95% CI, the significance level was set at p<0.05. Simple linear regression analysis was used to evaluate the 10-year incidence trend, allowing us to assess changes in incidence over time.

For prevalence calculations, results were stratified by year of birth in 5-year intervals to correct for potential biases of being included at a later date. The 10-year incidence rate for AN was calculated from the date of T1D diagnosis (or study entry for controls) up to 10 years of follow-up.

The proportional mortality ratio (PMR) was calculated by comparing the observed number of deaths in the study population to the expected number of deaths based on the control population. The formula used was:

PMR=(Observed number of deaths in study population)/(Expected number of deaths based on control population).

Kaplan-Meier estimators and log rank test were used to compare survival time from inclusion to diagnosis of AN in the T1D and control populations.

## Results

### Descriptive statistics

The study included 12 202 individuals (female n=5618) with T1D and 48 484 controls without T1D (female n=23 618). The mean age at inclusion was 9.6 years (SD=3.6) for both the T1D group and the matched control group (table 1). For the T1D group, inclusion date was defined as the date of diabetes diagnosis; for controls, the inclusion date was set to match the calendar date of their corresponding T1D case’s diagnosis date. This matching ensures comparable follow-up periods and age distributions between groups. The mean follow-up time was 150.6 months (approximately 12.5 years) for the T1D group and 151.4 months for the control group. In the T1D group, 0.9% (n=108, whereof 4 males) developed AN, compared with 0.6% (n=280, whereof 12 males) in the control group.

**Table 1:** Clinical characteristics

Characteristic	T1D cases, n=12 202		Controls, n=48 484	
	Female	Male	Female	Male
N (%)	5618 (46.0%)	6584 (54.0%)	23 618 (48.7%)	24 866 (51.3%)
Mean age at inclusion* (years±SD)	9.4±3.5	9.8±3.7	9.6±3.6	9.6±3.6
Mean age at follow-up (years±SD)	22.1±5.8	22.3±5.9	22.2±5.9	22.3±5.9
Mean age at AN diagnosis (years±SD)	16.9±3.9	15.8±2.1	16.6±3.7	16.2±3.9
AN (N, %)	104 (1.9%)	4 (0.06%)	268 (1.1%)	12 (0.05%)
Deaths (N, N due to AN)	44 (5)	63 (0)	46 (7)	119 (0)
Mean time from inclusion to AN diagnosis (years±SD)	7.2±4.6	6.8±2.4	7.1±4.7	6.9±4.2

* Inclusion refers to the time of T1D diagnosis for cases. For controls, the inclusion date was set to the same calendar date as their matched T1D case. Mean age at inclusion therefore reflects the age at the index date for both groups, which was determined by the T1D diagnosis date.

AN, anorexia nervosa; N, number of individuals; T1D, type 1 diabetes.

Since there were very few males with AN diagnosis, further analyses were performed in the female group only.

### Prevalence

The period prevalence of AN for females in the T1D group during the observed period was 1.9%, compared with 1.1% in the control group (table 2). The point prevalence of AN among 15-year-old females born between 1987 and 2005 was 0.87% for those with T1D and 0.53% for controls (incidence rate ratio (IRR) 1.65, 95% CI 1.16 to 2.35). For 20-year-old females born between 1987 and 2000, the point prevalence was 1.73% in the T1D population and 1.11% in the control population (IRR 1.55, 95% CI 1.20 to 1.99) (table 2).

**Table 2:** Prevalences and 10-year incidence of AN among female type 1 diabetes cases and female controls

	Type 1 diabetes cases, n=104	Controls, n=280	IRR (95% CI)
Period prevalence of AN	1.9%	1.1%	
Point prevalence of AN (15 years)*	0.87%	0.53%	1.65 (1.16 to 2.35)
Point prevalence of AN (20 years)†	1.73%	1.11%	1.55 (1.20 to 1.99)
10-year incidence of AN (per 100 000 person-years)	74.7	45.2	1.77 (1.35 to 2.32)

* Born 1987–2005.

† Born 1987–2000.

AN, anorexia nervosa; IRR, incidence rate ratio.

Compared with controls, the OR for developing AN among females with T1D across all birth cohorts was 1.64 (95% CI 1.31 to 2.06, p<0.001). When stratified by birth cohort, the highest OR (OR 2.36, 95% CI 1.48 to 3.75, p<0.001) was observed in females born between 1988 and 1992, indicating that females with T1D in this cohort had more than twice the odds of developing AN compared with matched female controls without diabetes (table 3).

**Table 3:** AN per birth cohort

Birth cohort	T1D no AN	T1D with AN	Control no AN	Control with AN	OR (95% CI)	P value
2003–2007	1327	9 (0.7%)	5552	26 (0.5%)	1.45 (0.68 to 3.10)	0.34
1998–2002	1501	36 (2.3%)	6364	90 (1.4%)	1.70 (1.15 to 2.51)	0.008
1993–1997	1414	24 (1.7%)	5781	76 (1.3%)	1.29 (0.81 to 2.05)	0.28
1988–1992	978	28 (2.8%)	4279	52 (1.2%)	2.36 (1.48 to 3.75)	<0.001
1982–1987	294	7 (2.3%)	1372	24 (1.7%)	1.36 (0.58 to 3.19)	0.48
Overall	5514	104 (1.9%)	23 350	268 (1.1%)	1.64 (1.31 to 2.06)	<0.001

Includes only females.

AN, anorexia nervosa; T1D, type 1 diabetes.

### Incidence

**Figure 1:**
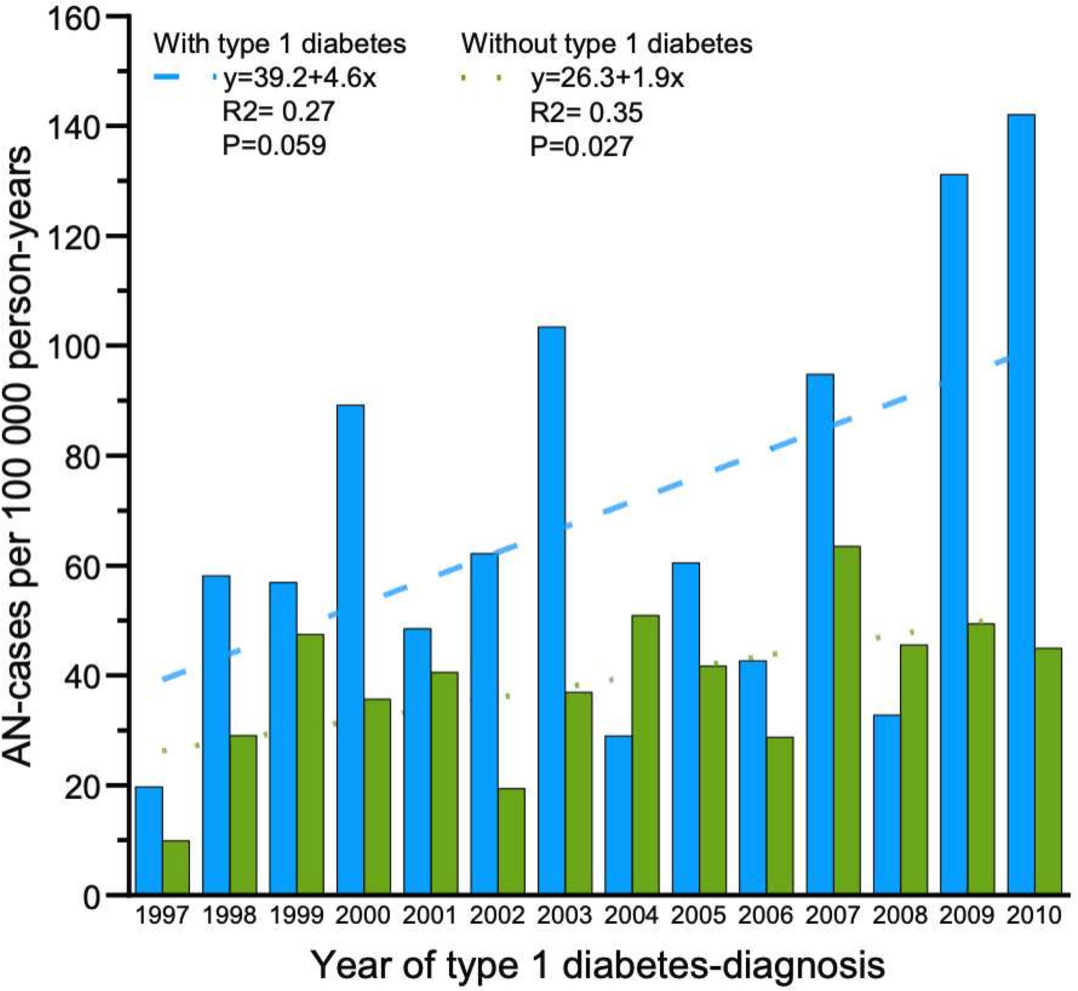
10-year incidence of AN in females with type 1 diabetes (blue bars) and in the control group (green bars), respectively. AN, anorexia nervosa.

The mean 10-year incidence rate of AN, from diagnosis of T1D and 10 years onward, was 74.7 per 100 000 person-years, compared with 45.2 per 100 000 person-years in the control group studied over the same time period (IRR 1.77; 95% CI 1.35 to 2.32) (table 2; figure 1).

The regression equation for the 10-year incidence trend in females with AN and T1D was: y=4.6 x+39.2 (R²=0.27, p=0.059) suggesting a weak and statistically non-significant linear relationship between the year and the incidence rate over the studied period (figure 1).

### Time to diagnosis

**Figure 2:**
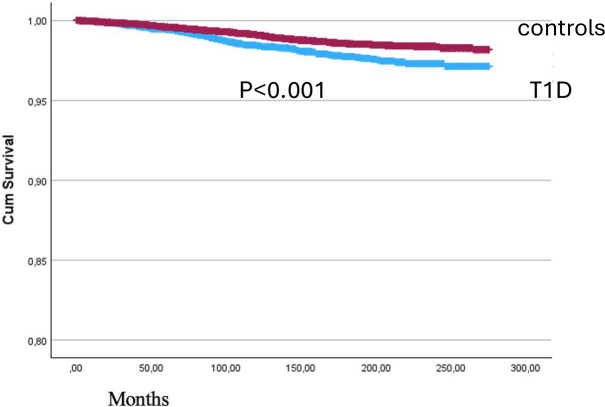
Kaplan-Meier survival curve displaying time to diagnosis of anorexia nervosa in T1D versus controls. T1D, type 1 diabetes.

The mean time from T1D diagnosis to AN diagnosis was 7.2±4.6 years for females and 6.8±2.4 years for males. The mean age at AN diagnosis was 16.9±3.9 years for females with T1D and 16.6±3.7 years for females without T1D (table 1). Kaplan-Meier survival analysis revealed a significant difference in the time to AN diagnosis between the T1D and control groups (log rank test, p<0.001; figure 2).

### Proportional mortality ratio

The PMR for females with both T1D and AN, compared with the T1D population without AN, was 4.5 (95% CI 1.5 to 10.4). Comparing the T1D+AN population to the non-diabetes AN group gave a PMR of 2.3 (95% CI 0.8 to 5.4). When comparing the T1D+AN population to the control population (without T1D and without AN), the PMR was 20.4 (95% CI 6.6 to 47.6) (table 4).

**Table 4:** PMR for females with AN and T1D

Comparison	PMR (95% CI)
AN versus control	8.3 (3.2 to 17.0)
T1D+AN versus T1D	4.5 (1.5 to 10.4)
T1D+AN versus AN	2.3 (CI 0.8 to 5.4)
T1D+AN versus control	20.4 (6.6 to 47.6)

Controls are without T1D and without AN. CI for PMR is based on Poisson distribution.

AN, anorexia nervosa; PMR, proportional mortality ratio; T1D, type 1 diabetes.

## Discussion

### Principal findings

This nationwide registry-based cohort study demonstrates that females with childhood-onset T1D in Sweden have a significantly elevated risk of developing AN compared with matched controls without T1D. Despite the elevated relative risk, the absolute risk of AN among females with T1D was 1.9%, reflecting that while risk is increased, most individuals with T1D do not develop AN. We found a 64% increase in the odds of AN and a 77% increase in the incidence rate. Most critically, the mortality burden is exceptionally high, with a PMR of 20.4 for females with both T1D and AN compared with controls without either condition. These findings have important implications for clinical practice, particularly regarding systematic screening and integrated care.

### Incidence of AN

The mean 10-year incidence rate of AN among females with T1D was 74.7 per 100 000 person-years compared with 45.2 per 100 000 person-years in controls. These rates are lower than the 580 per 100 000 person-years reported in a Finnish twin study by Silén *et al*,^16^ which used structured interviews and broader diagnostic criteria in a genetically enriched sample. The discrepancy likely reflects differences in case ascertainment methods; registry-based diagnoses capture clinically recognised cases requiring specialist care, whereas interview-based studies identify subclinical and unreported cases. It is well established that individuals with eating disorders, particularly AN, frequently do not seek medical attention.^17^ Therefore, our findings likely represent a conservative estimate of true AN occurrence.

The 10-year incidence trend showed a non-significant upward pattern (y=4.6 x+39.2; R²=0.27, p=0.059), suggesting possible increases over time. This trend may reflect improved detection and awareness of eating disorders in T1D populations, changes in diagnostic practices following the introduction of Diagnostic and Statistical Manual of Mental Disorders, Fifth Edition (DSM-5) criteria, or true increases in incidence. The critical clinical implication is the need for heightened vigilance, particularly given the specific risks associated with insulin omission for weight control.

### Prevalence of AN

The period prevalence of AN among females with T1D was 1.9% compared with 1.1% in controls. The control group prevalence aligns closely with the lifetime prevalence of 1.4% (range 0.1%–3.6%) for AN among females reported in a comprehensive systematic review by Galmiche *et al*^18^ supporting the validity of our findings. Point prevalence estimates at ages 15 and 20 years (peak risk periods) showed persistent elevation in the T1D group, with IRRs of 1.65 and 1.55, respectively.

Prevalence varied significantly across birth cohorts, with the highest prevalence (2.8%, OR 2.36 compared with non-diabetes cohort) observed in the 1988–1992 cohort. This peak during late adolescence and early adulthood is consistent with findings by Jones *et al*,^19^ who reported that 10% of adolescent females aged 12–19 years with T1D met diagnostic criteria for eating disorders compared with 4% of controls. The declining prevalence in more recent cohorts may reflect younger current age (insufficient follow-up time for AN onset),^20 21^ improved diabetes management strategies,^22^ or increased awareness and earlier intervention for T1D-eating disorder comorbidity.^23^ These findings align with a meta-analysis by Young *et al*,^11^ which reported a medium effect size for disordered eating behaviours in T1D adolescents and young adults.

Importantly, our study focused specifically on clinically diagnosed AN rather than the broader spectrum of eating disorders or disordered eating behaviours. Studies using screening instruments for disordered eating behaviours typically report higher prevalence estimates, as they capture subclinical presentations that may not meet full diagnostic criteria for AN.

### Mortality

The PMR of 20.4 for females with concurrent T1D and AN compared with controls without either condition represents a striking synergistic effect. For context, prior studies have reported standardised mortality ratios of approximately 5.9–6.2 for AN alone.^5 24^ Our study found a PMR of 8.3 for AN compared with controls, slightly higher than previous estimates, potentially reflecting the inclusion of more recent cohorts with ongoing follow-up. The PMR of 4.5 comparing T1D+AN to T1D alone demonstrates that AN substantially increases mortality risk even within the T1D population.

The mechanisms underlying this excess mortality are multifactorial. Insulin omission for weight control leads to chronic hyperglycaemia, accelerating microvascular and macrovascular complications.^25^ Nutritional deficiencies and electrolyte disturbances from AN increase the risk of cardiac arrhythmias. Psychiatric comorbidities, particularly depression, increase suicide risk.^7^ The combination of these factors creates a particularly lethal clinical syndrome requiring integrated psychiatric and endocrinological care.

### Time to diagnosis and age effects

The mean time from T1D diagnosis to AN diagnosis was 7.2 years for females, with mean age at AN diagnosis of 16.9 years in the T1D group vs 16.6 years in controls. Kaplan-Meier survival analysis revealed significantly shorter time to AN diagnosis in the T1D group, suggesting that T1D may accelerate AN onset or that the diabetes care environment creates specific triggers. This finding contrasts with Colton *et al*^26^ who reported similar ages of eating disorder onset in girls with and without T1D, but aligns with Nielsen *et al*,^25^ who observed earlier AN development in individuals with T1D. These discrepancies may reflect different study populations (Canadian vs Swedish) and diagnostic methods.

Existing literature indicates differing patterns of eating disorder onset and outcomes across developmental stages. Adolescence represents a particularly high-risk period due to the convergence of pubertal changes, intensified diabetes self-management demands and normative concerns about body image.^19^ Our cohort primarily reflects childhood-onset T1D with subsequent adolescent or young adult AN development.

### Sex differences

The overwhelming female predominance of AN in both the T1D group (96.3% female) and controls reflects the well-established sex distribution of AN. With only four male cases in the T1D group, our study lacked statistical power for sex-stratified analyses. However, the similar sex distribution in T1D and control groups suggests that T1D does not substantially alter the sex ratio of AN. Further research with larger samples is needed to examine potential sex-specific mechanisms and outcomes.

### Comparison with prior studies

Our findings extend and refine previous evidence. The Danish study by Dybdal *et al*^9^ reported that 1.5% of individuals with T1D received an eating disorder diagnosis over a mean follow-up of 7.8 years, slightly lower than our 1.9% period prevalence for AN specifically. This difference likely reflects our longer follow-up period (mean 12.5 years) and specific focus on AN. Early studies reporting no significant difference in AN prevalence between T1D and control populations^10^ were limited by small samples, methodological heterogeneity and data from periods when eating disorder awareness was lower. The present study, using comprehensive national registers with validated diagnoses and extended follow-up, provides more definitive evidence of elevated AN risk in childhood-onset T1D.

### Clinical implications

These findings strongly support routine screening for eating disorders in individuals with T1D, particularly adolescent and young adult females. Screening instruments such as the Diabetes Eating Problem Survey-Revised^27^ have been validated for this purpose. Early identification enables timely intervention and integrated care involving endocrinology, psychiatry, nutrition and psychology. Given the exceptionally high mortality ratio, clinicians should maintain high vigilance for warning signs including unexplained deterioration in glycaemic control, recurrent diabetic ketoacidosis and expressed concerns about weight or body image.

### Strengths and limitations

The main strengths include the large population-based sample, comprehensive national registry coverage with minimal loss to follow-up, validated diagnoses, extended follow-up period and matched control design. The validity of AN diagnoses in the Swedish National Patient Register has been formally assessed. Birgegård *et al*^28^ evaluated the diagnostic validity of eating disorder diagnoses in the NPR through chart review and found a positive predictive value of approximately 0.75 for AN diagnoses, indicating good diagnostic accuracy. The NPR has demonstrated high coverage and validity across multiple diagnostic categories,^29^ supporting the robustness of our findings.

Limitations include reliance on clinically diagnosed cases captured in specialist care registers, which likely underestimates true AN prevalence by missing cases managed exclusively in primary care or unreported cases. The low number of male cases limits generalisability to males. Registry data lack detailed clinical information on AN severity, treatment and recovery. We cannot determine temporal causality regarding whether specific aspects of T1D management contribute to AN development. Finally, our study focused on childhood-onset T1D; the relationship between adult-onset T1D/LADA and eating disorders may differ and warrants separate investigation.

### Conclusions and future directions

AN is significantly more prevalent and deadly in young females with childhood-onset T1D in Sweden compared with the general population. Despite elevated relative risk, absolute risk remains below 2%, indicating that most individuals with T1D do not develop AN. Nonetheless, the 20-fold increase in mortality for those with both conditions necessitates systematic screening and integrated care. Future research should focus on developing and evaluating targeted prevention and intervention programmes, identifying modifiable risk factors within diabetes care that may contribute to eating disorder development, and examining outcomes of integrated treatment models. Research on adult-onset T1D and eating disorders represents an important complementary area of investigation.

## Data Availability

Data are available on reasonable request.

## References

[1] Wei Y, Andersson T, Liu S (2025). Stable heritability of type 1 diabetes in a Swedish Nationwide Cohort Study. Nat Commun.

[2] Rawshani A, Landin-Olsson M, Svensson A-M (2014). The incidence of diabetes among 0-34 year olds in Sweden: new data and better methods. Diabetologia.

[3] Waernbaum I, Lind T, Möllsten A (2023). The incidence of childhood-onset type 1 diabetes, time trends and association with the population composition in Sweden: a 40 year follow-up. Diabetologia.

[4] Yao S, Kuja-Halkola R, Thornton LM (2016). Familial Liability for Eating Disorders and Suicide Attempts: Evidence From a Population Registry in Sweden. JAMA Psychiatry.

[5] Arcelus J, Mitchell AJ, Wales J (2011). Mortality rates in patients with anorexia nervosa and other eating disorders. A meta-analysis of 36 studies. Arch Gen Psychiatry.

[6] Falcão MA, Francisco R (2017). Diabetes, eating disorders and body image in young adults: an exploratory study about “diabulimia”. Eat Weight Disord.

[7] Eskild-Jensen M, Støving RK, Flindt CF (2020). Comorbid depression as a negative predictor of weight gain during treatment of anorexia nervosa: A systematic scoping review. Eur Eat Disord Rev.

[8] Blinder BJ, Cumella EJ, Sanathara VA (2006). Psychiatric comorbidities of female inpatients with eating disorders. Psychosom Med.

[9] Dybdal D, Tolstrup JS, Sildorf SM (2018). Increasing risk of psychiatric morbidity after childhood onset type 1 diabetes: a population-based cohort study. Diabetologia.

[10] Mannucci E, Rotella F, Ricca V (2005). Eating disorders in patients with type 1 diabetes: a meta-analysis. J Endocrinol Invest.

[11] Young V, Eiser C, Johnson B (2013). Eating problems in adolescents with Type 1 diabetes: a systematic review with meta-analysis. Diabet Med.

[12] National Guideline A (2017). National institute for health and care excellence: guidelines. eating disorders: recognition and treatment. london: national institute for health and care excellence (nice) copyright national institute for health and care excellence, 2017.

[13] Tate AE, Liu S, Zhang R (2021). Association and Familial Coaggregation of Type 1 Diabetes and Eating Disorders: A Register-Based Cohort Study in Denmark and Sweden. Diabetes Care.

[14] Eddy KT, Dorer DJ, Franko DL (2008). Diagnostic crossover in anorexia nervosa and bulimia nervosa: implications for DSM-V. Am J Psychiatry.

[15] Milos G, Spindler A, Schnyder U (2005). Instability of eating disorder diagnoses: prospective study. Br J Psychiatry.

[16] Silén Y, Sipilä PN, Raevuori A (2020). DSM-5 eating disorders among adolescents and young adults in Finland: A public health concern. Int J Eat Disord.

[17] Andersen ST, Linkhorst T, Gildberg FA (2021). Why Do Women with Eating Disorders Decline Treatment? A Qualitative Study of Barriers to Specialized Eating Disorder Treatment. Nutrients.

[18] Galmiche M, Déchelotte P, Lambert G (2019). Prevalence of eating disorders over the 2000-2018 period: a systematic literature review. Am J Clin Nutr.

[19] Jones JM, Lawson ML, Daneman D (2000). Eating disorders in adolescent females with and without type 1 diabetes: cross sectional study. BMJ.

[20] Ward ZJ, Rodriguez P, Wright DR (2019). Estimation of Eating Disorders Prevalence by Age and Associations With Mortality in a Simulated Nationally Representative US Cohort. JAMA Netw Open.

[21] Javaras KN, Runfola CD, Thornton LM (2015). Sex- and age-specific incidence of healthcare-register-recorded eating disorders in the complete swedish 1979-2001 birth cohort. Int J Eat Disord.

[22] Norman GJ, Fernandes J, Nemlekar P (2025). Initiating continuous glucose monitoring is associated with improvements in glycemic control and reduced health care resource utilization for people with diabetes in a large US-insured population: A real-world evidence study. J Manag Care Spec Pharm.

[23] O’Donnell NR, Satherley R-M, John M (2022). Development and Theoretical Underpinnings of the PRIORITY Intervention: A Parenting Intervention to Prevent Disordered Eating in Children and Young People With Type 1 Diabetes. *Front Clin Diabetes Healthc*.

[24] Papadopoulos FC, Ekbom A, Brandt L (2009). Excess mortality, causes of death and prognostic factors in anorexia nervosa. Br J Psychiatry.

[25] Nielsen S, Emborg C, Mølbak A-G (2002). Mortality in concurrent type 1 diabetes and anorexia nervosa. Diabetes Care.

[26] Colton PA, Olmsted MP, Daneman D (2015). Eating Disorders in Girls and Women With Type 1 Diabetes: A Longitudinal Study of Prevalence, Onset, Remission, and Recurrence. Diabetes Care.

[27] Wisting L, Frøisland DH, Skrivarhaug T (2013). Psychometric properties, norms, and factor structure of the diabetes eating problem survey-revised in a large sample of children and adolescents with type 1 diabetes. Diabetes Care.

[28] Birgegård A, Forsén Mantilla E, Dinkler L (2022). Validity of eating disorder diagnoses in the Swedish national patient register. J Psychiatr Res.

[29] Ludvigsson JF, Andersson E, Ekbom A (2011). External review and validation of the Swedish national inpatient register. BMC Public Health.

