# Supplementary material for: Incidence, prevalence and mortality of anorexia nervosa in individuals with childhood-onset type 1 diabetes: a nationwide retrospective cohort study in Sweden
Source: BMJ Open. 2026 Feb 12;16(2):e109015. doi: 10.1136/bmjopen-2025-109015 (PMC12911715; doi:10.1136/bmjopen-2025-109015)
Supplement: Supplementary file 1 [file bmjopen-2025-109015.reviewer_comments.pdf]

PEER REVIEW HISTORY

BMJ Open publishes all reviews undertaken for accepted manuscripts. Reviewers are asked to complete a checklist review form and are provided with free text boxes to elaborate on their assessment. These free text comments are reproduced below.

ARTICLE DETAILS

Title (Provisional)

Incidence, Prevalence, and Mortality of Anorexia Nervosa in Individuals with Childhood-onset Type 1 Diabetes: A Nationwide Retrospective Cohort Study in Sweden

Authors

Sjogren, Magnus; Englund, Erling; Erlandsson, Axel Åke, Elias; Möllsten, Anna

---

VERSION 1 - REVIEW

**Reviewer**	**1**
**Name**	**Tseng, Mei-Chih Meg**
**Affiliation**	**Taipei Medical University**
**Date**	**17-Sep-2025**
**COI**	**None**

The authors aimed to investigate the incidence, prevalence, and mortality of AN among patients with type I DM (T1D). This is a retrospective cohort study using a linkage between Swedish Childhood Diabetes Register (SCDR), Statistics Sweden, and National Patient Register. The study methodology is fine, and study findings are not new. The writing skill could have much room for improvement. Discussion could have included more explanations and elaborations regarding the higher prevalence and mortality of AN among patients with T1D than non-diabetics. Comments and suggestion are described as follows.

INTRODUCTION

The authors may provide the literature review regarding the epidemiology and association between AN and T1D in the INTRODUCTION to help readers understand what's the current state of knowledge about the research theme and what's the knowledge gap to be filled. To be deeper, the authors should examine the weakness of methodology or limitations in prior studies and highlight the value of this current study.

Research design and Methods

1. The authors did not mention any data censor, missing or covariates in statistics section.

Conclusion

1. A large part of the contents in Discussion could have mentioned in INTRODUCTION, including prior epidemiology study with comments on the different findings originated from using different methodology and among different samples.

2. The order of discussion for main findings may have been arranged more systematically. For example, to discuss the incidence first and then prevalence and mortality. Instead, the authors discussed the findings in a random order; paragraph 1 was about prevalence (p.11), 2 was about incidence (p.12), 3 was about prevalence (p.12), …and the paragraph was about the difference of incidence on p.14.

3. Some sentence was inappropriate. For example, while the authors mentioned "AN prevalence in the T1D group showed a decreasing trend in more recent cohorts, potentially due to younger age in more recent birth cohorts, but it may also be attributed to improved diabetes management strategies and increased awareness of T1S-eating disorder comorbidity." The following sentence "Our findings align with Young et al.'s..." was not relevant to the cohort topics.

**Reviewer**	**2**
**Name**	**Holmberg, Sara**
**Affiliation**	**Region Kronoberg, Department of Research and Development**
**Date**	**24-Sep-2025**
**COI**	**None**

Congratulations to a valuable study with clinically important findings based on high quality register data. I have some minor suggestions for revising the text.

1. Page 3 Twitter summary: "more common and deadlier": more than what? Please specify what comparison is intended. Other women? Other diseases? Other countries?

2. Page 4 keywords. Perhaps add eating disorder?

3. P 5 highlight. Point 1 do not answer to Why? Specify what is scientifically unknown or a clinical experience motivating the examination.

4. P 6, row 10. It would be valuable to include some numbers indicating how common T1D is. Perhaps is also valuable to mention that beside childhood onset T1D, adult onset is also common (for example: Thunander et al Incidence of type 1 and type 2 diabetes in adults and children in Kronoberg, Sweden. Diabetes Res Clin Pract. 2008 Nov;82(2):247-55.

This fact raises the question; what about patients with AN in younger ages developing T1D at a later stage. I recon that this is not a subject for this study but consider to comment on in the discussion

5. P 7, r 12. It is noted what is registred in the register but not what was included in the data set. Was it all children in the SCDR?

6. P 7, r 38. This sentence is unclear. Is it correct that all children and parents provided written informed consent for this specific study or was the consent for inclusion in the registry?

7. P 10, r 26-31. Specify what the OR's refer to.

8. P 10, r 56. "Fig 1" is abbreviated but spelled out on other places.

9. P 10, r 38. Is the heading “Conclusion” correct? Consider “Discussion”.

10. P 10, r 43. In this main finding consider adding that despite the high increased risk of AN in patient with T1D the absolute risk is still rather low.

11. Generally, in the discussion consider removing some numbers as repeating numbers from the result section is not needed. Focus on evaluating the results in relation to the literature which is adequately done.

12. P 12 r 52. “et al” is note cursive as in other places.

13. P 10-15. Consider elaborating somewhat more in the discussion on potential differences between children, adolescents and adults in accordance with the literature.

14. P 14, r 33. SD needs only one decimal. And this sentence is unclear, “mean age at diabetes onset” can not go for the control group. Please clarify. If the control group was well matched similar ages are expected. Is this what is commented on?

15. P 14, r 42. I question if it is correct to use the term “gender” if it is the biological sex that is referred to. Consider revising in the main text and in the abstract.

16. P 15, r 38. Consider adding some references to support the high validity of the Swedish diagnose registry.

17. Figure 2. Information on which color represents which group is lacking. Consider adding numbers and p-values to the figure or in the figure legend.

18. Page 26. Graphical abstract. The color coding is not clear and the prevalence numbers and stick figures therefore difficult to apprehend. Consider note which group is T1D and Control respectively.

VERSION 1 - AUTHOR RESPONSE

Response to Reviewer 1: Dr. Mei-Chih Meg Tseng
Introduction

- **Comment:** Expand literature review on the epidemiology and association between AN and T1D, addressing knowledge gaps and methodological weaknesses in prior studies.
  Response: The Introduction now provides an expanded review summarizing the state of knowledge, prior cohort and registry-based studies, the clinical overlap, limitations of self-report and small cohorts, and what remains unknown—especially regarding long-term mortality outcomes. The methodological strengths and weaknesses of prior work are highlighted.

Methods

- **Comment:** Address data censoring, missing data, and covariate use in the Statistics section.
  Response: Added a paragraph specifying rates of censoring (end of follow-up, loss to registry), description of handling missing outcome or covariate data with appropriate statistical techniques and sensitivity analyses.
  Replacement text:
  Handling of Missing Data and Censoring
  ”All individuals were followed from the date of T1D diagnosis (or corresponding

index date for controls) until first AN diagnosis, death, emigration, or end of the study period (December 31, 2019), whichever occurred first. Individuals lost to follow-up due to emigration or death were right-censored at the date of their last registered contact in Swedish national registers. The Swedish national registers have exceptionally high coverage (>99% for vital status and emigration), resulting in minimal loss to follow-up. Missing data on key covariates (age, sex, municipality) were absent due to the structure of the matching process. For clinical variables derived from the SCDR, coverage exceeded 98% for all key variables. Complete case analysis was therefore employed, and no imputation methods were used. Sensitivity analyses restricting to individuals with complete covariate data confirmed robustness of the main results (data available upon request)."

Discussion

- **Comment 1:** Move some prior study content to Introduction, especially prior epidemiology and methodological discussion.
  Response: Material on prior epidemiology studies and methodological considerations was moved to the Introduction.

- **Comment 2:** Rearrange the Discussion to discuss incidence, prevalence, then mortality, in a more systematic order.
  Response: The Discussion now presents the principal findings in this recommended order.
- **Comment 3:** Clarify sentences regarding decreasing prevalence and make reference linking explicit for each finding. Ensure references are relevant and not misplaced.
  Response: The relevant paragraph have been updated and clarified with appropriate references. See revised Discussion section.

Response to Reviewer 2: Dr. Sara Holmberg

1. **Twitter summary:**

- **Comment:** Clarify what "more common and deadlier" is compared to.
  Response: Now reads: " Anorexia nervosa is significantly more common and deadlier in females with type 1 diabetes compared to age- and sex-matched females without diabetes in Sweden. Routine screening for eating disorders is recommended."

2. **Keywords:**

- **Comment:** Add 'eating disorder' to keywords.
  Response: Added.

3. **Article highlight point 1:**

- **Comment:** Specify unknowns or clinical reasoning leading to the study.
  Response: Highlights removed. In accordance with Journal guidelines.

4. **Prevalence of T1D in Sweden; adult-onset T1D:**

- **Comment:** Provide data on T1D prevalence and note adult-onset T1D (citing Thunander et al.).
  Response: Added:

" In Sweden, approximately 1,000 children are diagnosed with type 1 diabetes annually, corresponding to a cumulative incidence approaching 0.9% by age 18 years in recent birth cohorts (Wei et al., 2025), …"

5. **Description of SCDR inclusion:**
- **Comment:** Clarify included subjects from SCDR.
  Response: Added statement in Methods: " All children diagnosed with T1D before age 15 years and registered in the Swedish Childhood Diabetes Register (SCDR) from July 1, 1977 onwards were included."

6. **Consent statement clarification:**
- **Comment:** Clarify if consent was for the study or for registry.
  Response: This has been clarified: " At initial registration in the Swedish Childhood Diabetes Register, informed consent was obtained from children and their parents for inclusion in the register. Swedish legislation permits the use of national register data for research purposes under ethical committee approval without requiring renewed individual consent from participants and thus, no additional consent was necessary nor obtained for the present study."

7. **Odds Ratio clarification:**
- **Comment:** Specify ORs and reference groups.
  Response: Each OR is now clearly stated with reference (e.g., "Compared to controls, the OR for AN in females with T1D was…").

8. **Figure abbreviation consistency:**
- **Comment:** Ensure use of "Fig" or "Figure" is consistent.
  Response: Uniform abbreviation ("Figure") used throughout.

9. **Heading 'Conclusion'/'Discussion':**
- **Comment:** As per earlier editor comment, corrected globally.

10. **Absolute risk context:**
- **Comment:** Add that absolute risk remains low despite higher relative risk.
  Response: Statement added to main finding: " Despite the elevated relative risk, the absolute risk of AN among females with T1D was 1.9%, reflecting that while risk is increased, most individuals with T1D do not develop AN."

11. **Repetitive numbers in discussion:**
- **Comment:** Limit numbers in Discussion; focus on interpretation.
  Response: Discussion streamlined to avoid unnecessary repetition of numerical data. However, when deemed relevant, numbers have been kept.

12. **Formatting 'et al.':**
- **Comment:** Ensure italicized formatting for "et al."
  Response: Corrected throughout the manuscript.

13. **Children/adolescent/adult group diYerences:**
- **Comment:** Discuss possible dioerences between these groups.
  Response: revised and now reads: Prevalence varied significantly across birth cohorts, with the highest prevalence (2.8%, OR 2.36 compared to non-diabetes cohort) observed in the 1988-1992 cohort. This peak during late adolescence and early adulthood is consistent with findings by Jones et al. {Jones, 2000 #16257},

who reported that 10% of adolescent females aged 12-19 years with T1D met diagnostic criteria for eating disorders compared to 4% of controls. The declining prevalence in more recent cohorts may reflect younger current age (insuoicient follow-up time for AN onset) {Ward, 2019 #16399} {Javaras, 2015 #16400}, improved diabetes management strategies {Norman, 2025 #16401}, or increased awareness and earlier intervention for T1D-eating disorder comorbidity {O'Donnell, 2022 #16402}. These findings align with a meta-analysis by Young et al. {Young, 2013 #16258}, which reported a medium eoect size for disordered eating behaviors in T1D adolescents and young adults."

14. **SD decimal and mean age at diabetes onset:**

- **Comment:** Use 1 decimal for SD; clarify age data for control group.

  Response: Tables and text now use one decimal (e.g., "Mean age at diabetes onset was 9.6 years [SD 3.6] for both T1D and control groups, where controls were assigned the same index year.")

15. **Use sex vs. gender:**

- **Comment:** Revise to 'sex' where biological sex is meant.
  Response: All references to gender now use 'sex' per ICD-10 conventions.

16. **Validity of diagnosis in NPR:**

- **Comment:** Add references on Swedish NPR's validity for AN and EDs.
  Response:
  New reference: Birgegård A et al. Validity of eating disorder diagnoses in the Swedish National Patient Register. J Psychiatr Res. 2022;150:227-230. https://doi.org/10.1016/j.jpsychires.2022.03.057 [Positive predictive value for AN ~0.75]

17. **Figure 2 legend:**

- **Comment:** Clarify color coding; add group designations and p values.
  Response: Figure legend now includes color codes, group labels, and a note about statistical significance.

18. **Graphical abstract:**

- **Comment:** Clarify color coding and group allocation; clarify prevalence values visually.
  Response: Graphical abstract updated per request (see revised figure submission for details).

Additional References Added

- References for all new literature and registry validity are provided above and in the revised reference list, including PubMed/DOI links as required.

VERSION 2 - REVIEW

**Reviewer**	**2**
**Name**	**Holmberg, Sara**

Affiliation **Region Kronoberg, Department of Research and Development**

Date **18-Nov-2025**

COI

The manuscript has been well revised in accordance with comments. The graphical abstract is however still a bit confusing. Why are the stick figures three boys and one girl? This does not reflect the data. The synergistic effect could perhaps be more pronounced with another layout.
